# Supplementary material for: Lithocholic Acid Induces miR21, Promoting PTEN Inhibition via STAT3 and ERK-1/2 Signaling in Colorectal Cancer Cells
Source: Int J Mol Sci. 2021 Sep 22;22(19):10209. doi: 10.3390/ijms221910209 (PMC8508661; doi:10.3390/ijms221910209)
Supplement: Supplementary file 1 [file ijms-22-10209-s001.zip › ijms-1330295-supplementary.pdf]

Supplementary data

Lithocholic acid induces miR21, promoting PTEN inhibition via STAT3 and ERK-1/2 signaling in colorectal cancer cells

Thinh-Thi Nguyen[1,2†], Thuan-Trong Ung[1,2†], Shinan Li[1], Dhiraj Kumar Sah[1], Sun-Young Park[1], Sen Lian[3*] and Young-Do Jung[1*]

[1]Research Institute of Medical Sciences, Chonnam National University Medical School, Gwangju 501-190, Republic of Korea
[2]Nanogen Pharmaceutical Biotechnology Joint Stock Company, Ho Chi Minh City 71207, Vietnam.
[3] Department of Biochemistry and Molecular Biology, School of Basic Medical Sciences, Southern Medical University, Guangzhou 510515, Guangdong, China

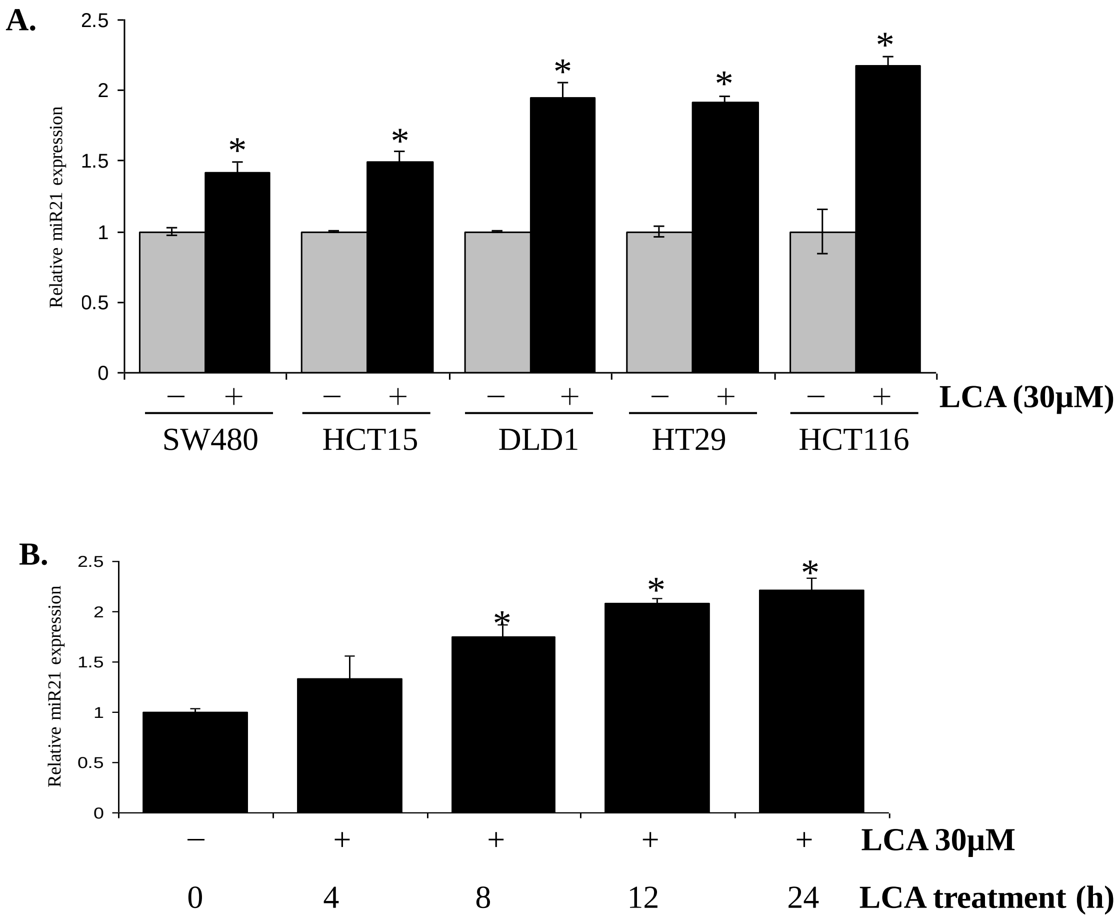

Figure S1. LCA induces miR21 expression in colorectal cancer cell lines. **(A)** Different colorectal cancer cell lines were treated with 30μM LCA for 24h and assessed miR21 expression by Realtime-PCR. **(B)** HCT116 cells were treated with 30μM LCA for different time periods and then checked miR21 expression by Realtime-PCR. * $p < 0.05$ versus control. The above data represent the means ± SD from triplicate measurements.

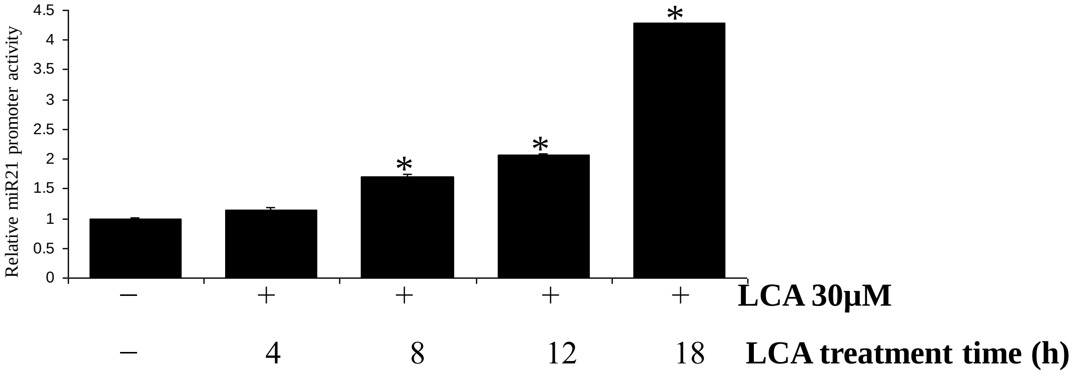

Figure S2. LCA stimulated miR21 promoter activity by time dependence. pGL3-miR21 promoter transfected HCT116 cells were treated with 30μM LCA for 4h−18h and then checked for miR21 promoter activation by dual-luciferase assay. * $p < 0.05$ versus control. The above data represent the means ± SD from triplicate measurements.

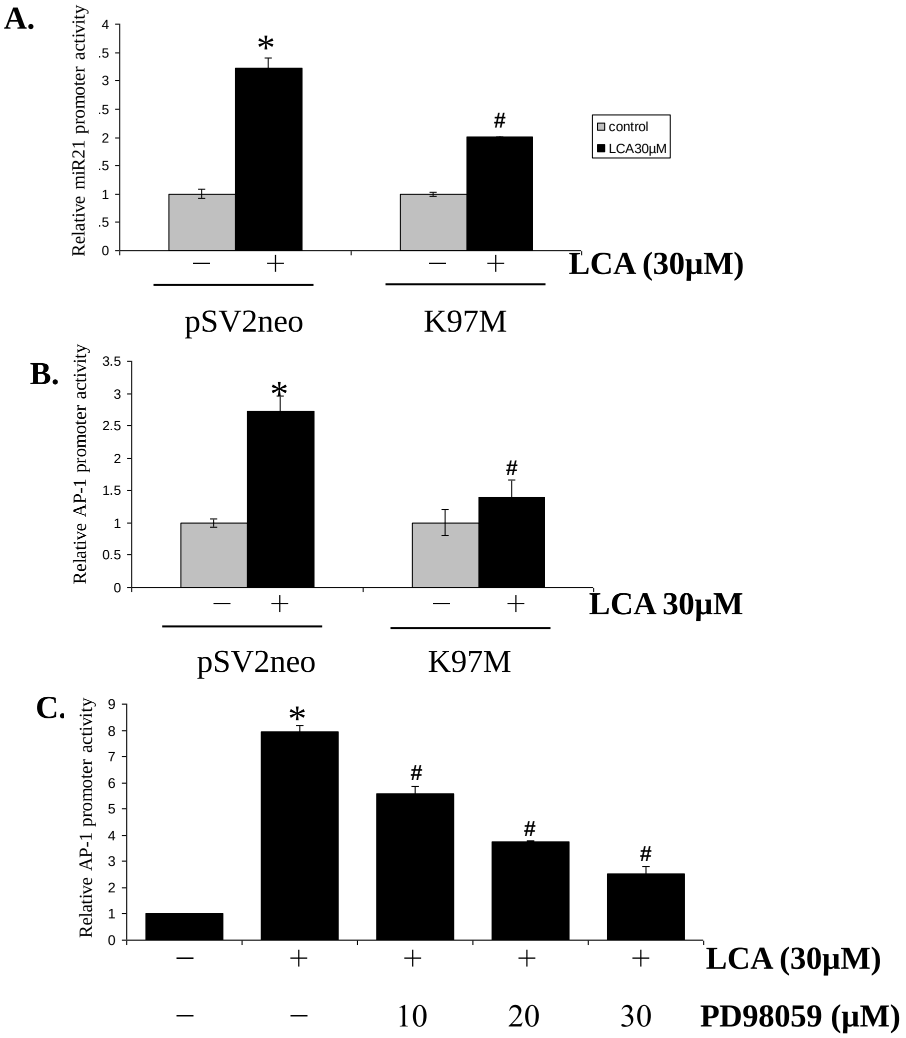

Figure S3. Erk1/2 signaling is involved in LCA-induced miR21 through AP-1 transcription factor activation. (A) The dominant mutant of Erk1/2 (K97M) or pSV2neo (1μg) were co-transfected into HCT116 cells with 250ng of pGL3-miR21 promoter. The cells were then treated with 30μM LCA for 18h and subjected for miR21 promoter activity checking by dual-luciferase assay. **(B)** The dominant mutant of Erk1/2 (K97M) or pSV2neo plasmid (1μg) were co-transfected into HCT116 cells with 250ng of pGL3-AP1 promoter. The cells were then treated with 30μM LCA for 18h and subjected for AP1 promoter activity checking by dual-luciferase assay. (**C**) HCT116 cells transfected with pGL3-AP1 promoter were treated with PD98059 at 10μM−30μM for 1h and then 30μM LCA for 18h. The cells were then harvested, lysed and checked for AP-1 promoter activity by dual-luciferase assay. *: $p < 0.05$ versus control; #: $p < 0.05$ versus only LCA. The above data represent the means ± SD from triplicate measurements.12

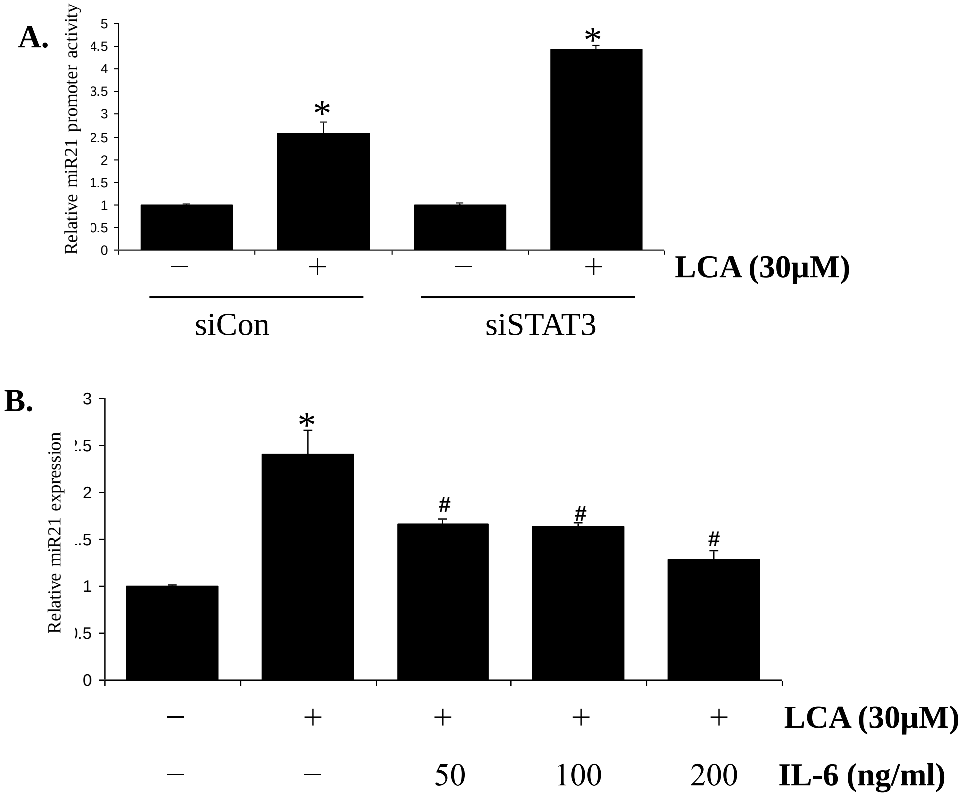

Figure S4. STAT3 signaling is involved in LCA-induced miR21 expression in HCT116 cells. (A) HCT116 cells transfected with siSTAT3 or siCon (50nM) were further transfected with pGL3-miR21 promoter. The cells were then treated with 30μM LCA for 18h and subjected for miR21 promoter activity by dual-luciferase assay. **(B)** HCT116 cells were treated with IL-6 at 50ng/ml−200ng/ml for 1h prior to LCA treatment for 24h. The cells were then harvested to extract total RNA and checked for miR21 expression. * $p < 0.05$ versus control; #$p < 0.05$ versus only LCA. The above data represent the means ± SD from triplicate measurements.

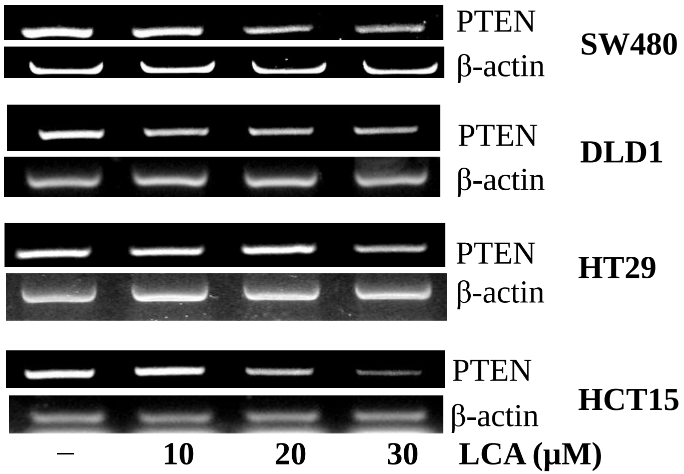

Figure S5. LCA inhibits PTEN expression in CRC cell lines. Different CRC cell lines treated with 10μM–30μM LCA within 24h were checked for PTEN expression by RT-PCR**.**

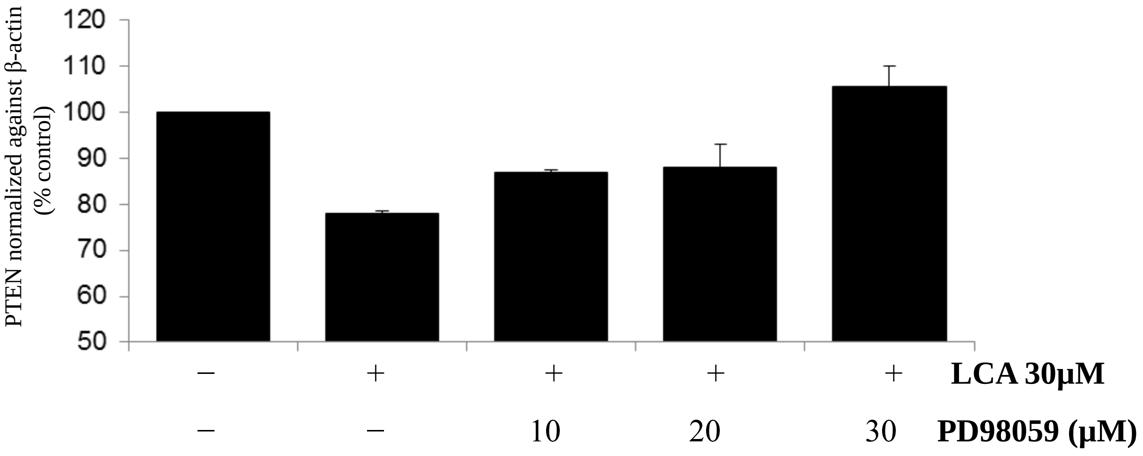

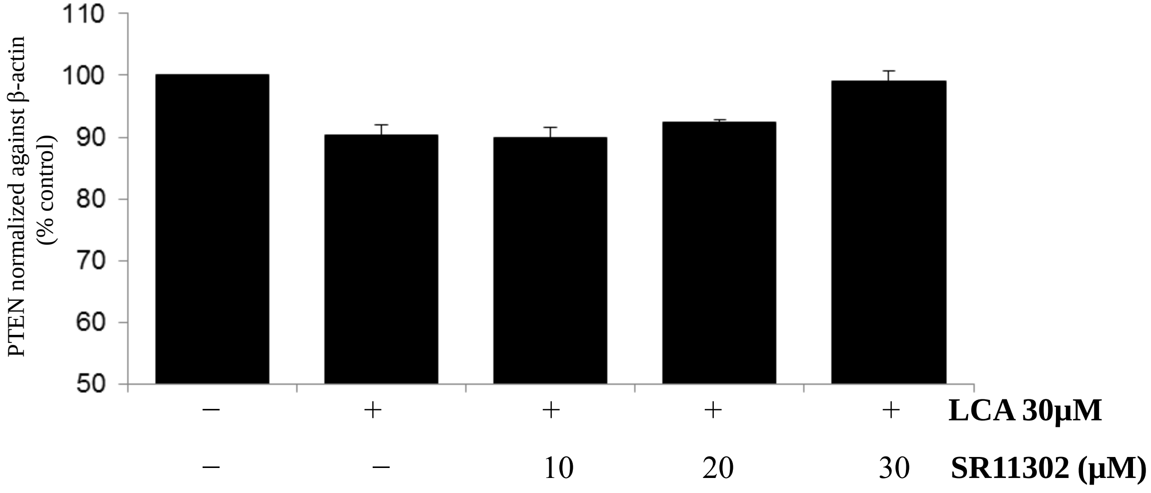

Figure S6. Densitometric WB analysis of Figure 6B (A) and 6D (B). The above data represent the means ± SD from different experiments.
